# Supplementary material for: Engineering of Silica Mesoporous Materials for CO2 Adsorption
Source: Materials (Basel). 2023 Jun 4;16(11):4179. doi: 10.3390/ma16114179 (PMC10254491; doi:10.3390/ma16114179)
Supplement: Supplementary file 1 [file materials-16-04179-s001.zip › materials-2301796-supplementary.pdf]

Engineering of silica mesoporous materials for CO_2 adsorption

Oyundari Tumurbaatar[1], Margarita Popova[1], Violeta Mitova[2], Pavletta Shestakova[1] and Neli Koseva[3*]

[1]Institute of Organic Chemistry with Centre of Phytochemistry, Bulgarian Academy of Sciences, Acad. G. Bonchev St., bl. 9, 1113 Sofia, Bulgaria;

[2]Institute of Polymers, Bulgarian Academy of Sciences, Acad. G. Bonchev St., bl. 103A, 1113 Sofia, Bulgaria;

[3]Bulgarian Academy of Sciences, №1, 15 Noemvri St., 1040 Sofia, Bulgaria;

* Correspondence: koseva@polymer.bas.bg; Tel.: +359887297361

Supplementary Material

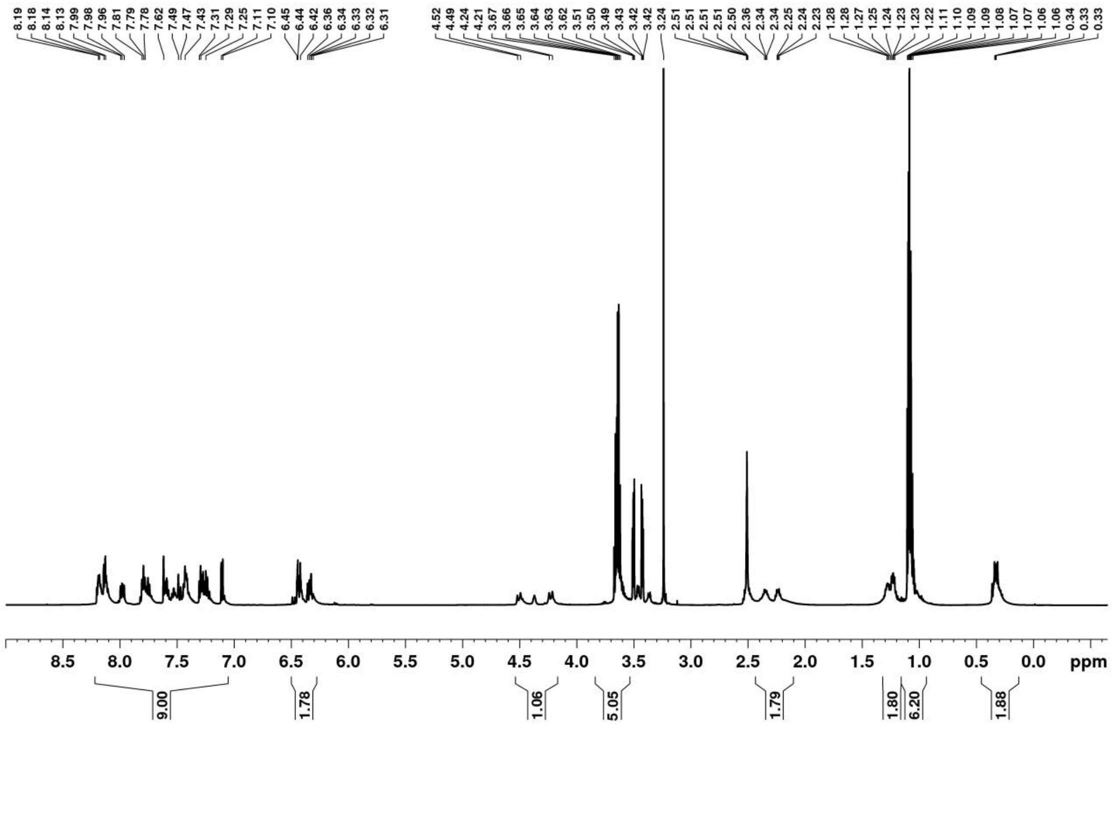

Figure S1. ^{1}H NMR spectrum of DAPTES in DMSO-d6.

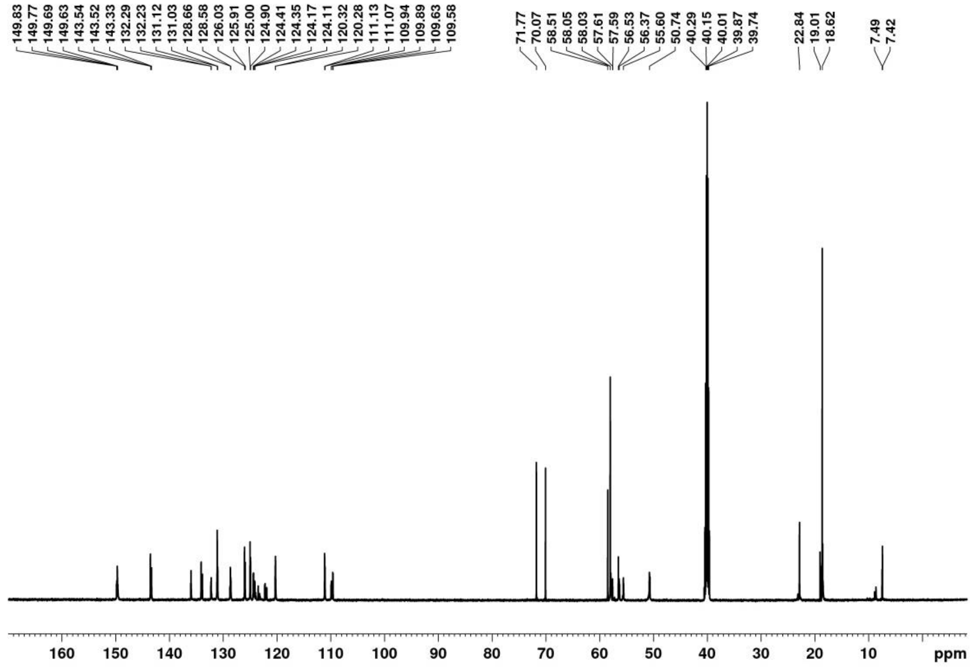

Figure S2. ^{13}C NMR spectrum of DAPTES in DMSO-d6.

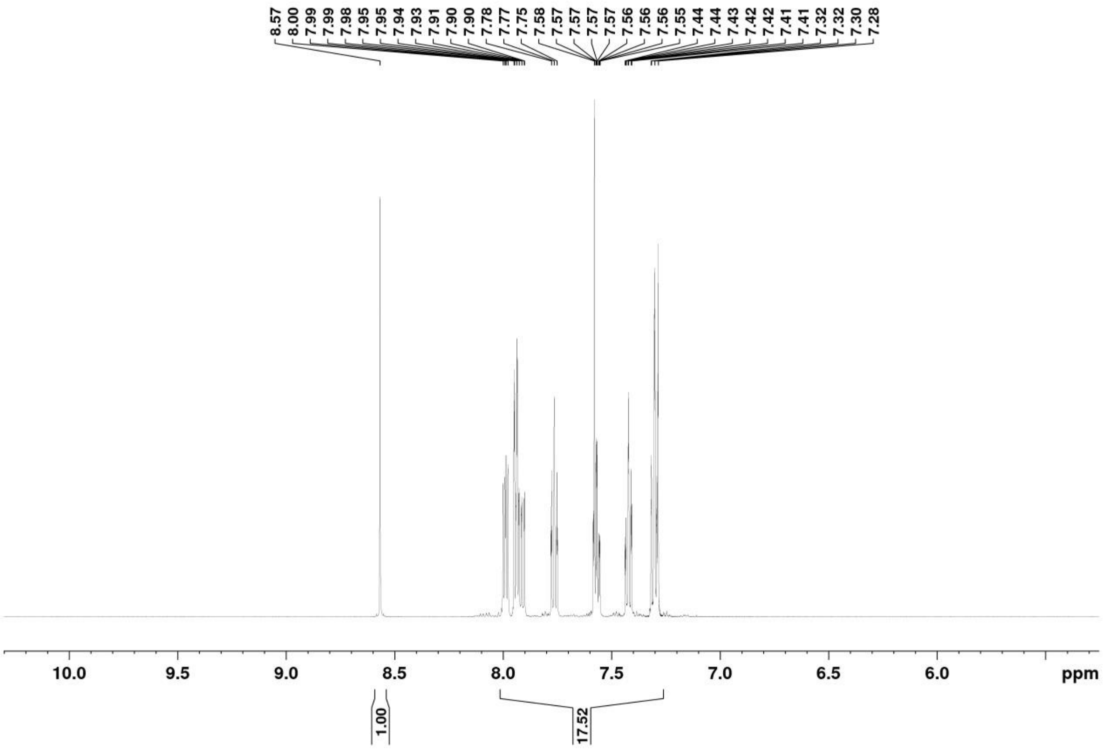

Figure S3. ^{1}H NMR spectrum of DOPO in $CDCl_3$.

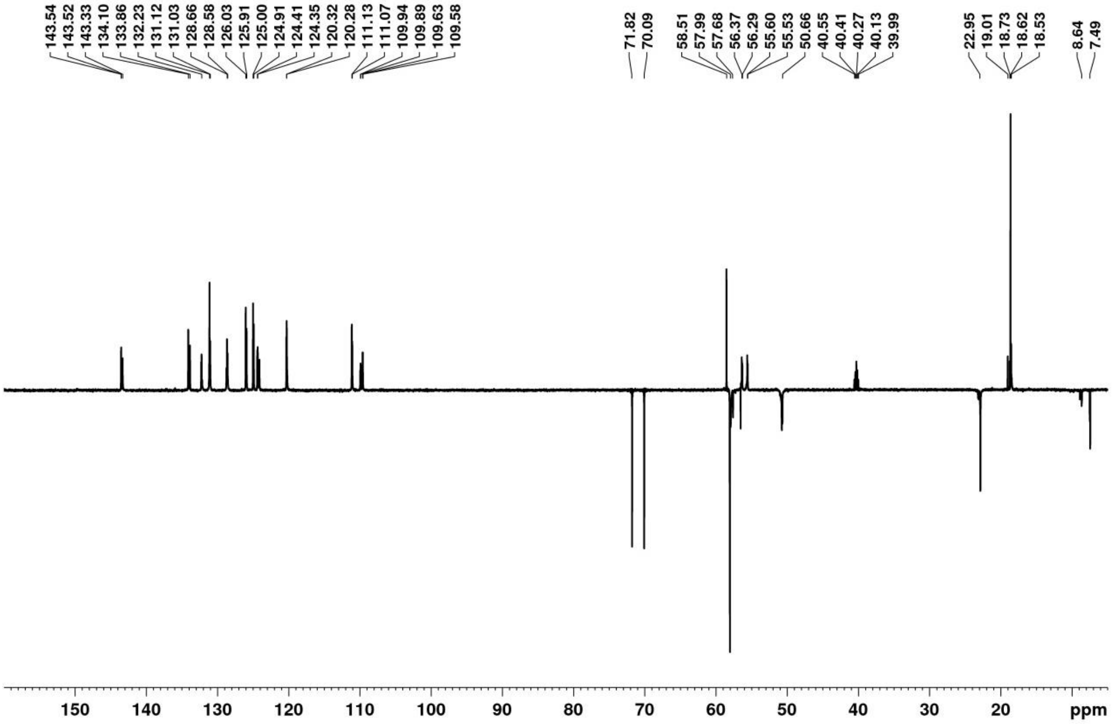

Figure S4. DEPT-135 NMR spectrum of DAPTES in DMSO-d6.

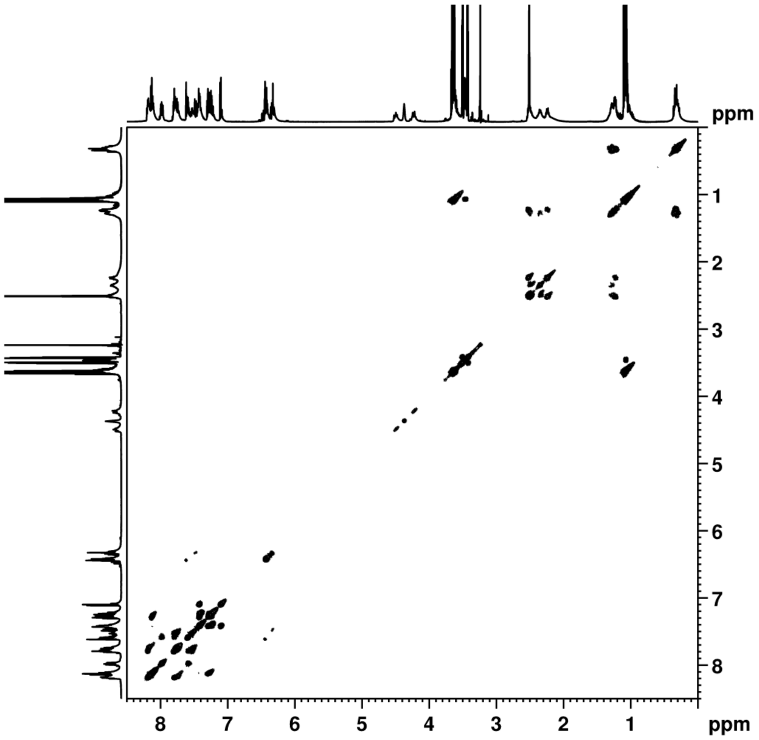

Figure S5. COSY spectrum of DAPTES in DMSO-d6.

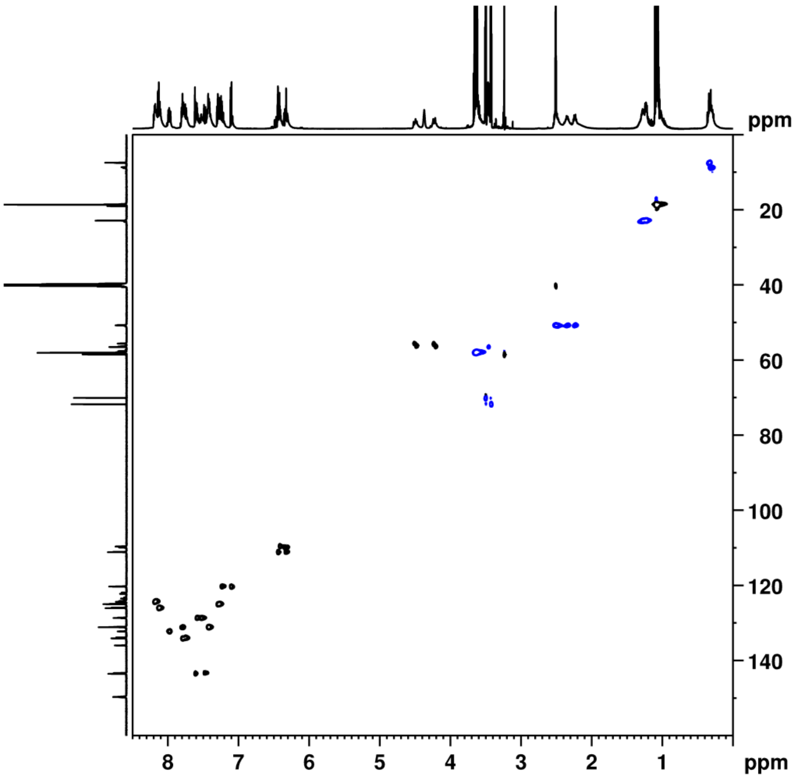

Figure S6. ^{1}H-^{13}C HSQC spectrum of DAPTES in DMSO-d6.

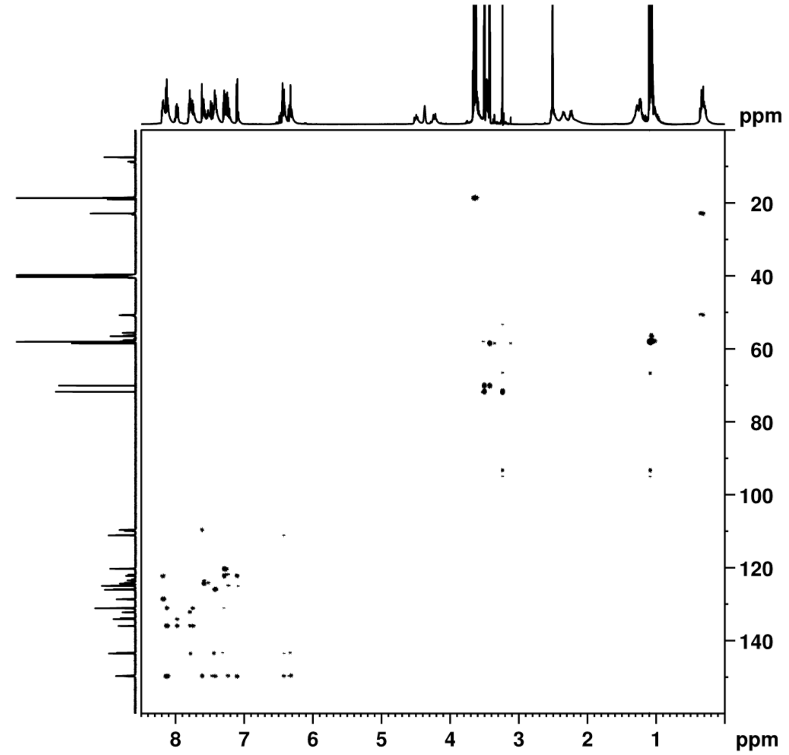

Figure S7. ^{1}H-^{13}C HMBC spectrum of DAPTES in DMSO-d6.

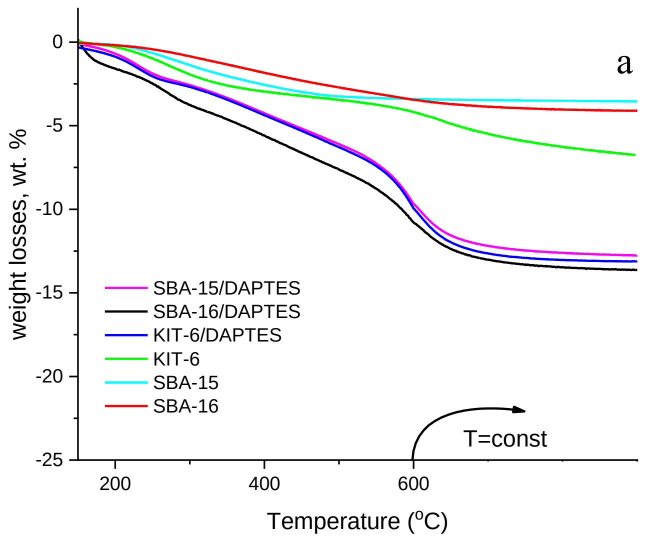
a
0
-5
-10
-15
-20
-25
200
400
600
weight losses, wt. %
Temperature (°C)
SBA-15/DAPTES
SBA-16/DAPTES
KIT-6/DAPTES
KIT-6
SBA-15
SBA-16
T=const

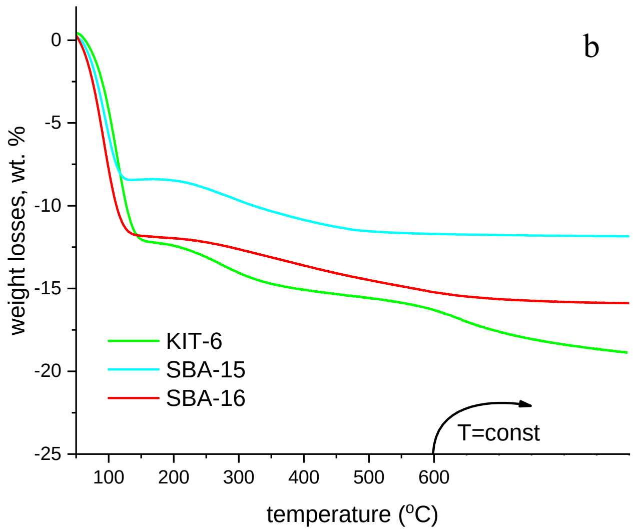
b
0
-5
-10
-15
-20
-25
100
200
300
400
500
600
weight losses, wt. %
temperature (°C)
KIT-6
SBA-15
SBA-16
T=const

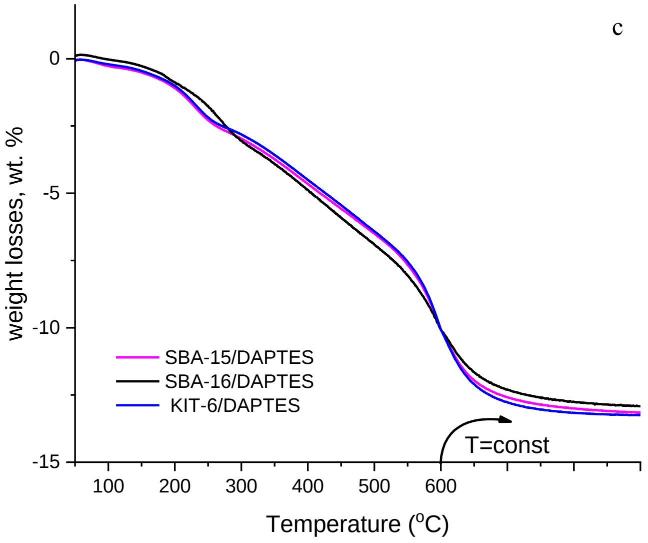

Figure S8. a) TG curves of the initial and the DAPTES-modified silicas in the temperature range 150 ℃ – 600 ℃ used to determine the weight loss due to decomposition of the grafted moieties;

b) TG curves of the initial silicas in the temperature range 50 ℃ – 600 ℃. The weight loss up to 120 ℃ – 130 ℃ is due to adsorbed humidity;

c) TG curves of the modified silicas in the temperature range 50 ℃ – 600 ℃. The weight loss at 150 ℃ is less than 0.5% which is evidence for the hydrophobization of the pore surface as a result of DAPTES grafting and the thermal stability of the DAPTES moieties.

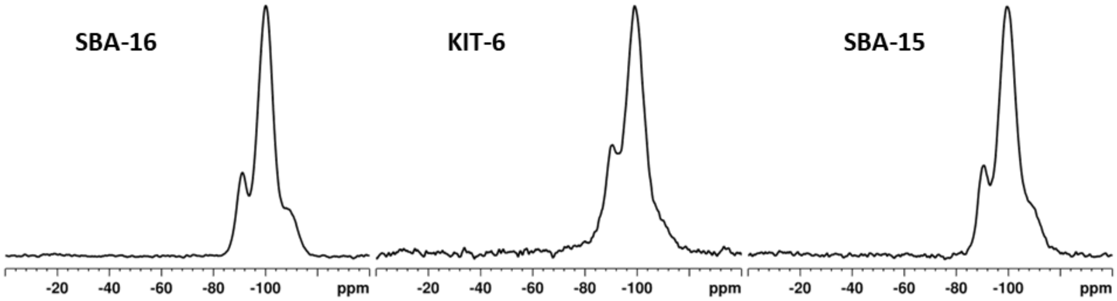

Figure S9. $^{1}H \rightarrow ^{29}Si$ CP MAS spectra of parent SBA-16, KIT-6 and SBA-15.

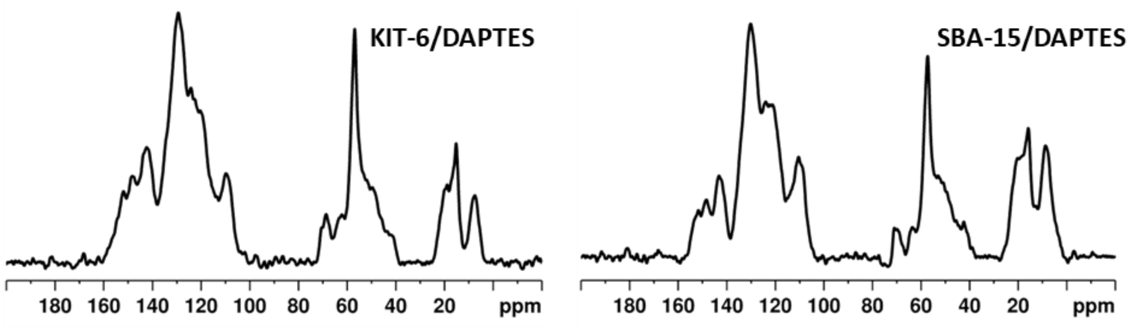

Figure S10. $^{1}H \rightarrow ^{13}C$ CP MAS spectra of KIT-6/DAPTES and SBA-15/DAPTES samples.

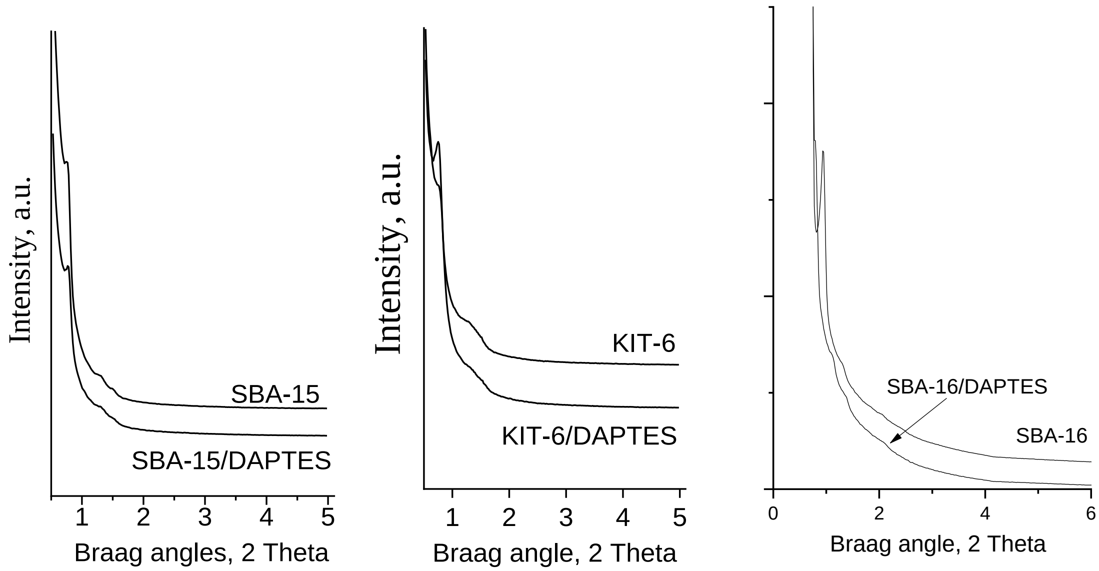

Figure S11: Low angle XRD patterns of the SBA-15, KIT-6 and their DAPTES-modified analogs

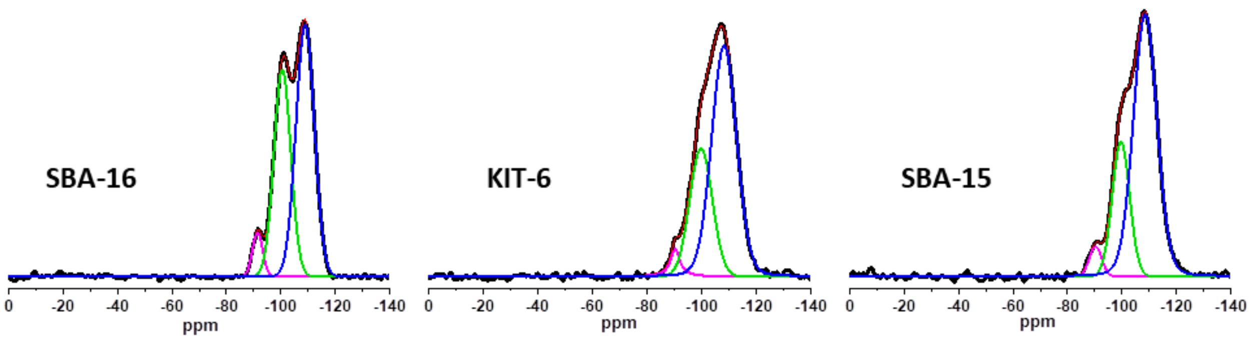

Figure S12. Experimental (black) and simulated (red) single-pulse ^{29}Si NMR spectra of the a) SBA-16, b) KIT-6, c) SBA-15. The individual contributions of the different Si environments

obtained by the deconvolution of the spectral patterns are given with colored lines (Q^4 - blue, Q^3 - green, Q^2 - magenta).

The results from the deconvolution of the spectra with DMFit software [D. Massiot, F. Fayon, M. Capron, I. King, S. Le Calve, B. Alonso, J.O. Durand, B. Bujoli, Z. Gan, G. Hoatson, Modelling one- and two-dimensional solid-state NMR spectra. Magnetic Resonance in Chemistry 40 (2002), 70-76] are summarized in Table S1.

Table S1. Area of the signals of the different Si structural units, obtained by deconvolution of the spectral patterns in the quantitative single pulse 29Si NMR spectra of the parent mesoporous silica materials.

Sample	Q^4 (Si,0OH) -108 ppm	Q^3 (Si,1OH) -100 ppm	Q^2 (Si,2OH) -90 ppm
SBA-16	54	**41**	5
KIT-6	65	**31**	4
SBA-15	70	**26**	4

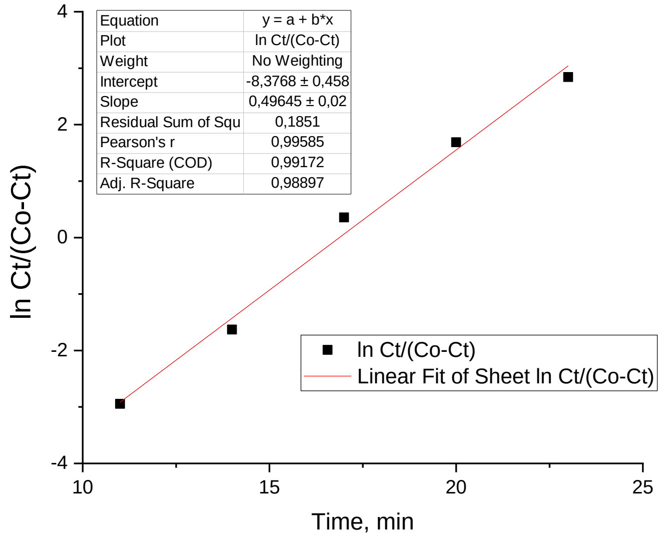

Figure S13. Fitting of experimental data on kinetic model at 0 ◦C.
